# Porous Bead-Based Diagnostic Platforms: Bridging the Gaps in Healthcare

**DOI:** 10.3390/s121115467

**Published:** 2012-11-09

**Authors:** Jie Chou, Jorge Wong, Nicolaos Christodoulides, Pierre N. Floriano, Ximena Sanchez, John McDevitt

**Affiliations:** 1 Department of Bioengineering, Rice University, 6100 Main St MS-142, Houston, TX 77005, USA; E-Mails: jie.chou@rice.edu (J.C.); christo@rice.edu (N.C.); pfloriano@rice.edu (P.N.F.); vsan@rice.edu (X.S.); 2 Department of Chemistry, University of Texas at Austin, 1 University Station A5300, Austin, TX 78712, USA; E-Mail: jwongc@gmail.com; 3 Department of Chemistry, Rice University, 6100 Main St MS-142, Houston, TX 77005, USA

**Keywords:** point-of-care, beads, porous, immunoassays, microfluidics, clinical chemistry, validation

## Abstract

Advances in lab-on-a-chip systems have strong potential for multiplexed detection of a wide range of analytes with reduced sample and reagent volume; lower costs and shorter analysis times. The completion of high-fidelity multiplexed and multiclass assays remains a challenge for the medical microdevice field; as it struggles to achieve and expand upon at the point-of-care the quality of results that are achieved now routinely in remote laboratory settings. This review article serves to explore for the first time the key intersection of multiplexed bead-based detection systems with integrated microfluidic structures alongside porous capture elements together with biomarker validation studies. These strategically important elements are evaluated here in the context of platform generation as suitable for near-patient testing. Essential issues related to the scalability of these modular sensor ensembles are explored as are attempts to move such multiplexed and multiclass platforms into large-scale clinical trials. Recent efforts in these bead sensors have shown advantages over planar microarrays in terms of their capacity to generate multiplexed test results with shorter analysis times. Through high surface-to-volume ratios and encoding capabilities; porous bead-based ensembles; when combined with microfluidic elements; allow for high-throughput testing for enzymatic assays; general chemistries; protein; antibody and oligonucleotide applications.

## 1. Introduction

Diagnostic tools are critical to the delivery of effective healthcare treatment, yet current *in vitro* diagnostic (IVD) devices to date have been shown to be incapable of keeping pace with the rapidly increasing information content related to disease diagnosis and progression generated with advanced “omics” methods such as genomics, proteomics, metabolomics and glycomics [1,2]. Here, despite the thousands of biomarker discovery papers published, only 1.5 protein biomarkers per year on average have received US FDA approval during the past 15 years [3–6]. Unfortunately, most modern clinical analyzers are dedicated to single classes of analytes and are burdened by bulky, expensive, laboratory-confined instrumentation preventing broad access to these assays at the point-of-care (POC). The movement of new technologies to POC settings and the use of noninvasive sampling modalities have important implication in terms of improvement in the efficiency of the delivery of healthcare. Unfortunately, to date POC devices suffer in two major respects relative to their remote laboratory counterparts. First, in general the POC devices are more expensive and, second, these portable systems more often than not, yield performance inferior to that derived from traditional laboratory settings [7,8]. Furthermore, large sample volume requirements and lack of standard instrumentation that is responsive to a broad range of analytes complicate clinical validation studies that need to follow the initial discoveries and proof of principle phases.

Traditional approaches to clinical analysis involve a well-appointed centralized laboratory, three degrees of separation from the patient. This hierarchy introduces a number of critical junctures in which errors may be introduced and delays incurred. To simplify and offer assay results immediately, research into devices that give results at the POC, specifically bedside, ambulance or remote location, currently flourishes—a situation advantageous to both patients and healthcare providers [9–13]. POC diagnostic systems have been extensively reviewed in recent years, from both the points of view of usage [14–16] and fabrication [17–19]. The ability to process large amounts of information at the point-of-need is common in the field of electronics, yet the ability to similarly process complex molecular disease signatures has not yet been fully demonstrated [7]. The marriage of microelectronics and IVD areas provides huge opportunities to healthcare industries seeking affordable and accessible diagnostic infrastructures [7,20].

In the past few decades, significant advances in medical microdevice technologies have afforded new sensor ensembles capable of multiplexed detection of a wide range of analytes [21–23], including diagnostic targets, such as disease-specific proteins [15], metabolites and other small molecules [24], nucleic acids [25–27], bacteria and bacterial spores [28–34], and human cells [19,35,36]. Diagnostic devices for limited-resource settings, including the developing world, have seen significant development efforts recently as this area requires new affordable technologies that can work outside of the traditional laboratory settings [19,36–41].

Through the miniaturization of macro-components, micro total analysis system (μTAS) and lab on a chip (LOC) devices have ushered in a new generation of high-throughput testing modalities that promise new options for biomarker measurements [7,42–44]. For example, Quake's work has advanced the “large-scale integration” of microfluidics, analogous to the electronics field [20]. Mirkin, Heath and Wang used nanowires, precious metal nanoparticles, and magnetic techniques, respectively, to measure diverse sample types and create a variety of assembly types [13,45,46], while Sia has introduced more integrated approaches via microelectromechanical systems [47]. Singh has continued to increase integration through the use of chip-based separation and quantitation [48]. Both Singh and Ligler have extended their integrated approaches into the rapid, multiplexed detection of toxins and other biothreats [49]. Work by Madou and others have resulted in the LabCD, which eliminates traditional active mechanical valves and pumps by using centrifugal and centripetal force to perform fluid movement and control [50]. Walt's work with electronic noses uses arrays of optical fibers as the underlying infrastructure for biological sensing systems [51]. Finally, researchers in the Toner group have explored a number of novel methods for the isolation and enumeration of lymphocytes, erythrocytes, and circulating tumor cells [52,53].

There now is a strong potential to leverage these medical microdevice discoveries for a broad impact in diagnostics for IVD and global heath applications using such chip-based approaches. Unfortunately, to date very few complete workable POC clinical devices have emerged despite tremendous progress in LOC, microfabrication, microfluidics, and related areas [43,54]. Indeed, while the core of typical LOC systems is substantially smaller than that of the bench-top counterparts, most systems still rely on a network of macroscopic laboratory-based infrastructure for sample processing, sample introduction, analyte detection, data processing, and reagent handling, thus limiting their utility for POC applications [7].

In addition to key work in the LOC area, which includes on-chip sample processing, significant progress has also been made recently in the area of protein, antibody and oligonucleotide planar microarray technologies with off-chip (*i.e.*, lab confined) sample processing. The emergence of high-density planar microarrays has enabled parallelized testing for clinical testing and validation. With modest sample volume requirements, these microarrays have afforded multiplexed testing of hundreds to thousands of analytes simultaneously for both proteomic and genomic applications [55–59]. Unfortunately, the cumbersome and time-consuming processing steps, as well as the large expense of the microarray disposable elements, have limited their utility to sophisticated research settings. Thus, the microarray systems that are now popular in research venues have not yet impacted significantly routine clinical settings [60].

To overcome these limitations with respect to time course, sampling and cost, recent advancements in the integration of bead sensors into microfluidic devices have demonstrated short timeframes for analysis that brings promise for their use at the point-of-care [61,62]. These highly sensitive sensing elements have attracted significant interest for the detection of biological and chemical agents for applications ranging from cardiac and cancer health, drug screening, and environmental screening [63–66]. Compared to the gold standard, enzyme linked immunosorbent assay (ELISA), which takes 2 to 24 h to complete, analyses with bead sensors can be completed in less than an hour [64,67]. Further, the multi-functionality of these beads introduced many possibilities for their incorporation into microfluidic devices for the detection of a wide range of analytes. Low nonspecific binding properties, highly parallelized processes for production, and multiplexing capabilities of beads offer significant new opportunities for use in the context of near-patient testing.

This review article examines the convergence of LOC technologies with bead-based sensor ensembles with biomarker validation alongside the use of porous media, so as to service the high-fidelity capture and analysis of a plurality of key biomarker systems in validated studies. While several prior reports have summarized the advantages of solid-state bead supports [61,67–69], the use of porous beads has not been covered previously in great detail. As summarized here, the enhanced mass transport, tunable porosities and high binding capacities of porous beads serve as key variables that have strong potential to lead to transformative changes in high-performance biomarker detection using scalable detection approaches. The combination of these elements allows for the development and deployment of high-performance platform systems that can function for the first time at the POC with performance that rivals the traditional remote laboratory instrumentation.

## 2. Solid-State Bead Sensors

### 2.1. High Surface-To-Volume Ratio

Many modern bioscience analyte detection approaches such as ELISA utilize flat surfaces to generate signals. These approaches are limited by the low intrinsic signaling capabilities and slow transport characteristics afforded by these low-dimensional systems, which in most cases also rely on time-consuming amplification strategies. When compared to flat surfaces, 3-dimensional spherical beads offer significantly higher surface areas for immobilization of capture probes. For example, calculations by Kawaguchi show that 1 g of microspheres with a diameter of 0.1 μm has a surface area of 60 m^2^ [67]. With the same mass, further decreases in bead size would increase total surface area. Flat surfaces, however, are constant and limited to the open surface area available on the device. In contrast to the performance of flat surface-based immunoassays, the higher available surface area on beads increases sensitivities and lowers limits of detection [70].

When beads are interfaced with microfluidics, convective flow replenishes the analytes that become bound to capture probes. In contrast, assays performed with ELISA are for the most part static aside from some modest agitation where the dominant method for transport is diffusion. Because the diffusion distance is a few millimeters and the time to diffuse a distance is proportional to the square of the specific distance, ELISAs typically require several hours to overnight to perform [42]. The high surface-to-volume ratio of beads allows for timeframes of bead-based assays to be compressed relative to these planar counterparts.

When microfluidics is integrated with bead-based capture ensembles, immunoassays can be performed over much shorter timeframes. For example, Sato *et al.* revealed the surface-to-volume ratio of 45 μm polystyrene beads was ∼37 times higher than that of the flat surface in a microtiter plate [71]. Because of the high surface-to-volume ratios and the short diffusion distances afforded by the trapping of beads at the end of a barrier, the equilibration time for the capture of human secretory immunoglobulin A on beads was found to be 90 times less than that on flat surfaces. Likewise, the total analysis time was reduced from 24 h to less than 1 h. Similarly, Zammatteo *et al.* compared the capture of DNA probes on the surfaces of polystyrene microwells and beads [72]. When the total surface area on the wells was equal to that on beads (1.4 cm^2^), the final signal was found to be similar. However, the incorporation of higher amounts of beads increased the total surface area for capture. In contrast, the total surface area for binding on microwells remained constant. By increasing the amount of beads per test by a factor of 4, the signals on microbeads were two times more than those of microwells. Moreover, nucleic acid hybridization kinetics performed much faster on beads than on microwells.

### 2.2. Plug and Play

Further, beads exhibit a high degree of practicality with respect to their production and incorporation into microfluidic devices. The synthesis of beads can be completed in a very scalable manner. Beads can be produced in bulk quantities on the order of millions to billions of beads per batch. Once made, the surfaces (and interior regions in the case of porous media) of such beads allow for the functionalization of a variety of a capture probes for both genomic and proteomic applications. The beads produced and functionalized in these batches exhibit high reproducibility for both size and chemistry considerations. Moreover, beads produced in such large numbers benefit from the economics of scale with lower cost associated to each sensor. Once processed, these beads can be stored for long durations (*i.e.*, several years) until they are ready for use [7]. Multiple sets of beads functionalized with different capture probes can be quickly inserted into microfluidic devices as “plug and play” elements to address different clinical needs.

In contrast, functionalization of capture probes on a planar microarrays is done through a very serialized, tedious, and time-consuming process [69]. For example, passive immobilization of antibodies usually requires several hours due to the slow rate of diffusion of free probes. During the attachment of capture probes, further quality assurance measures must take place to ensure the same immobilization conditions on surfaces treated with the same chemistry. Because beads are functionalized in batches, the statistical difference from bead-to-bead is very low. Further, a modification of an array necessitates the creation of new devices and a microarray configuration. With beads, a modification of a test panel is often as simple and an addition, subtraction, or replacement of a bead with different functionalized probes [70,73]. Functionalized beads can be inserted into chip structures within seconds using “pick and place” strategies adapted from the microelectronics industry. The ability to complete quality control on a large batch of beads in a highly parallel manner once, instead of completing similar oversight for every device that is generated by alternative spotting or lithographic reagent deposition steps, serves as a huge potential advantage for the bead sensor ensemble approaches and provides testament for the growing interests and popularity of the bead-based approaches in the bioscience and clinical measurement fields [7,74,75].

### 2.3. High-Throughput

Often in clinical tests, the amount of sample is limited, such as is the case with neonatal testing. Further, prospective clinical trials and animal studies serve as additional areas where the completion of testing with minimal sample volume is critically important. Therefore, the ability to test for multiple analytes in a single sample simultaneously allows for efficient and faster results with the use of less expensive reagents and limited sample volumes. Microarray techniques have allowed for high-throughput testing in this capacity. Through the spotting of different capture probes on planar surfaces, multiple analytes can be detected simultaneously as mentioned above. Each location is spatially encoded to detect a specific type of target analyte. Spatially recognizable software can decode and quantify each test location. Delehanty *et al.* used a microarray printer to spot antibodies on discrete locations within 6 channels of a glass slide. This development led to the simultaneous detection of both protein and bacterial analytes [76].

Similarly, bead sensors can be incorporated in large quantities into microfluidic devices to allow for highly parallelized detection of analytes and samples. The use of multiple beads to target a specific type of analyte permits statistical redundancy for high quality analyses. The multiplexing of beads functionalized with different capture probes can similarly be performed with minimal work [77,78]. For example, Ng *et al.* revealed that the incorporation of an array of polymer beads held by micropillars allows for the spatially addressable, rapid detection of nucleotides and multiple bacterial species [79]. This methodology afforded the capacity for DNA-based detection of 10 bacterial species and 2 single nucleotide polymorphisms in less than 10min. In addition, Zhao *et al.* used encoded photonic beads to simultaneously profile the biomarkers CA125, CA19-9, AFP, and CEA associated with tumors including colorectal, gastric, and lung cancer [80]. The use of silica colloidal crystal beads allowed for the identification of the four different bead types. Furthermore, the Walt group uses 3 μm, spectrally encoded polymer beads for detection of numerous targets [81–83]. This high-throughput platform can detect around 100 different DNA targets simultaneously with highly statistical precision through high redundancy of each bead types.

Additionally, in the remote laboratory setting the integration of beads with suspension array technology (SAT) allows for rapid sample processing with rates in the thousands of measurements per second [84]. This platform decodes and measures encoded beads, typically only a few microns in diameter, in a flow cytometer. The combination of microbeads and flow cytometry technology can process through a 100-plex assay every 30 s [85]. Under a continuous automated process, this system can analyze almost 300,000 assays each day. Further, Kuckuck *et al.* demonstrated that the rate of processing can be further increased to 96-well plates per minute [86]. Here, the throughput limit approached the rate of the autosampler. SATs have evolved to accept beads for both applications in proteomics and genomics [87]. For example, SATs have allowed the high-throughput testing of pathogenic diseases [88], cytokines [89], and nucleic acid [90].

### 2.4. Encoding

In multiplexed assays in planar microarrays, the identities of capture probes are determined from their positions in the array. This positional encoding affords microarrays the ability to perform thousands of tests simultaneously. Similarly, methods to attach a code to each bead (encode) allows for its differentiation (decode) from other bead types and permits parallel screening of multiple analytes in a single sample.

One of the most common methods to encode beads is to employ a fluorophore. These luminescent dyes with different spectral characteristics and concentration values allow for a set of uniquely distinguishable codes. These spectrally encoded beads are commonly used in flow cytometers. Luminex Corp, one of most well-established bead-based instrument suppliers, uses three fluorophores to encode a panel of up to 500 different 5.5 μm beads. Each bead type is matched to a specific capture probe. Using a 2-laser system, beads delivered through a suspension array are quickly decoded and their intensities are measured [91]. Similarly, BD Biosciences offers fluorescently dyed 7.5 μm beads of different concentrations [55,65,68,92]. With a two-laser system, these beads are analyzed inside the BD FACSArray which has multiplexed capabilities to detect up to four different spectral wavelengths [55]. A 96-well plate containing processed samples can be analyzed at a rate of 15,000 events per second. The use of these spectrally encoded beads on these flow cytometry-based platforms have been demonstrated for the detection of single nucleotide polymorphisms [93], cytokines [94–96], bacterial pathogens [97,98], and infectious diseases [99]. Several studies, performed on these systems, reported analyses times much shorter than that of ELISA with sensitivities and increased dynamic ranges that compare or rival ELISA [85,100,101].

**Figure 1.**
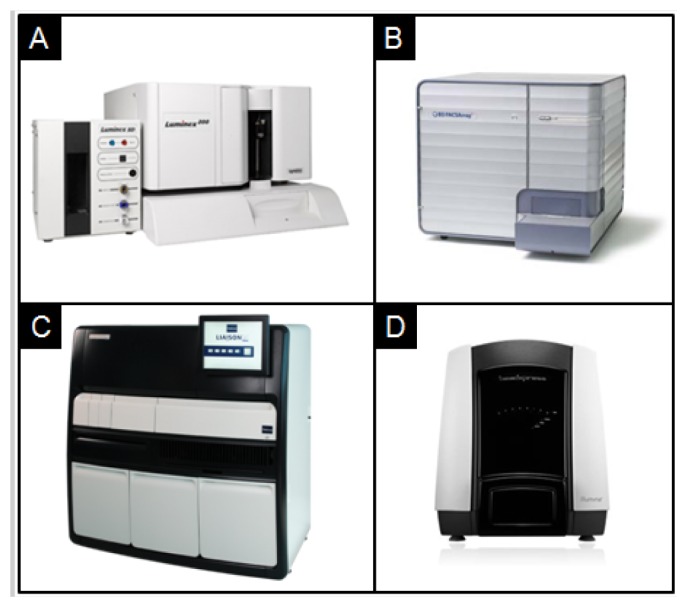
Current solid-state bead-based clinical laboratory analyzers include the (**A**) Luminex 100/200, (**B**) BD FACSArray, (**C**) Diasorin Liaison, and (**D**) Illumina BeadXpress.

Similarly, Illumina developed a high-density optical fiber microwell array [81,102–104]. The tips of these glass optical fibers are etched with hydrofluoric acid to create a 5 μm well. When bundled together, this array contains 50,000 fibers with a diameter of 1–2 mm [102]. When immersed in a solution of spectrally encoded beads, tens of thousands of 3 μm beads randomly disperse and assemble onto the etched microwell array. After excess solution and microspheres are removed, an imaging system decodes and quantifies the signal on each bead. The microarray has a test density that is significantly higher than that on an automatically spotted planar microarray. Because of this high density, only a small volume of sample is required to run tens of thousands of tests in a single run. Similarly, Illumina has also developed an etched silicon chip containing a hexagonal array of microwells, each measuring ∼3 μm, that can hold randomly dispersed beads. Using a CCD camera, individual beads are decoded and quantified. BioArray Solutions, which was purchased by Immucor, Inc. in 2008, uses a similar technology as Illumina. In this BeadChip format, encoded beads are randomly patterned onto a silicon chip, for the detection of complex nucleic acids and proteins [105,106]. Figure 1 showcases some of the current nonporous bead-based clinical analyzers that are used for remote laboratory measurements.

One limitation of these encoding schemes is the complication of possible overlap between spectral encoders or reporters. This complication limits the potential amount of simultaneous tests that can be performed in a single assay to about 100 different groups. The positionally-addressable identification of bead types, analogous to location-based encoding in microarrays, may extend the limit of different tests performed in a single assay. For example, Ng *et al.* used an array of polyacrylamide gel pads to form pillars to trap different bead types. Because of the natural immobilization of beads to the polymeric matrix, beads with similar probes are easily anchored in gaps between the micropillars. A second set of beads with a different probe set, spotted onto different positions of the array, allow for the differentiation of bead types. This process can be repeated to attach different beads [79,107]. Similarly, the Ikami *et al.* immobilized microbeads in hydrogel supports to allow for position-based, addressable decoding [108]. Here, fluid containing microbeads with similar capture probes is photo-polymerized with a photomask to form a hydrogel pillar. Uncured solution is washed. The process is repeated for beads with different probe sets. This bead-hydrogel device allows for the simultaneous detection of three proteins in about 4 min using only 0.5 μL of total sample and reagents. Other less common approaches to encode beads include chemical, graphical, electronic, and physical encoding [69].

## 3. Towards Point-of-Care

The advantages of high-throughput multiplex testing through high surface-to-volume ratios of solid support beads have allowed for shorter analysis times with low sample and reagent requirements. Nonetheless, the timeframes to complete these tests are often still not consistent with the POC [109,110]. For example, a typical doctor's visit that lasts 15–30 min does not permit for a diagnostic test that requires more than 1hr to complete. Previously, a set of guidelines for POC tests has been developed and designated with the acronym **COMMAND QUALS** [75]. Likewise, clinical analyzers need to be **C**heap, **O**bvious, **M**iniaturized, **M**ultiplexed, **A**utomated, **N**onperishable, **D**ependable, **Q**uick, **U**nobtusive, **A**daptable, **L**imited (volume), and **S**elf-contained.

Improvements in mass transport and high-efficiency signaling are crucial here to achieve the ideal timeframes and high-fidelity analyte detection using simple instrumentation as is necessary in POC usage. Advantages of enhanced mass transport in porous mediums, such as gel pads and hydrogels, have demonstrated faster timeframes over planar microarrays. With the ability to functionalize a range of different capture probes, these porous networks have the ability to capture a wide range of analytes. The development of new microstructure concepts with engineered active transport through and within porous reactive particles serves as a promising new method for rapid yet high-efficiency capture within minisensor ensembles [111–114]. The ability for analytes to transport into the interior matrix and high capacities for capture probes have allowed for shorter analysis times and higher sensitivities than those of planar microarrays [115,116].

**Figure 2.**
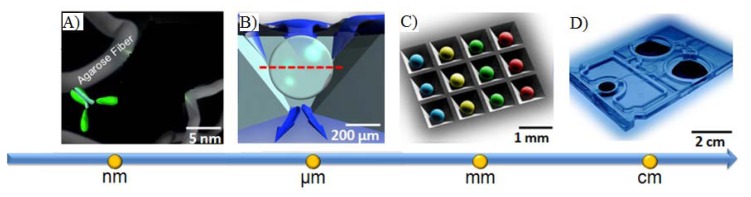
(**A**) Nanonets created from agarose fibers serve as a high surface area medium onto which can be attached a high-density of biocapture agents such as antibodies, (**B**) these nanonets comprise the backbone of a microsponge (*i.e.*, porous bead) unit that is both indexed matched to the aqueous medium as well as serving as the location for analyte capture and reporting, (**C**) the beads are arrayed in predefined locations within a chip structure that is being developed to feature integrated microfluidic delivery elements all supported within (**D**) Photograph of an injection-molded lab card dedicated to CD4 counting. Specifications with lab cards with identical footprint are being developed with laminate approaches and customized to contain all of the fluids, reagents and self-contained waste provisions for the bead-based application.

In the supported porous bead array area, prior efforts have led to the development of a microfluidic bead-based platform termed the Programmable Bio-Nano-Chip (p-BNC). This p-BNC platform is based on the concept that multiplex assays for a variety of disease applications could be achieved on a modular sensor suite that combines an application specific cartridge in conjunction with a universal analyzer. The approach uses a sensing platform that employs an array of 280 μm porous, agarose beads situated in an array combining individual flow-through wells. As shown in Figure 2, fluid containing analytes of interest flow through and around the porous bead sensors that are situated in individual flow-through containers. Delivered analytes are captured by immobilized capture probes within the bead matrix and can be quantified with a secondary fluorescently labeled reporter. The unique p-BNC design provides enhanced convective transport to the interior of porous bead matrices, efficient diffusion distances, and short depletion layers [117]. The use of porous beads in this pressure-driven design results in increased sensitivities compared to those of flat microfluidic channels.

**Figure 3.**
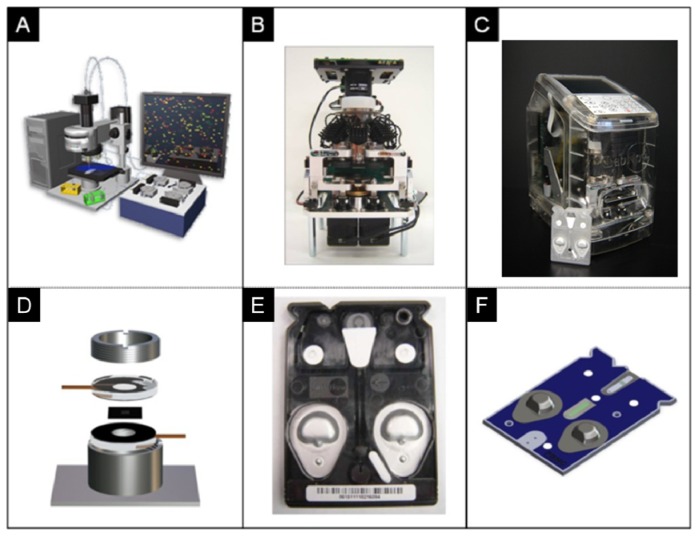
The evolution of several generations of the p-BNC. (**A**) Starting with a laboratory benchtop configuration, the p-BNC consists of an array of anisotropically etched microarrays on a silicon microchip held within a stainless steel flow cell. These systems are now moving through rigorous clinical testing (see below). (**B**) Previous translational efforts with commercial firms have defined target specifications for evolution from the benchtop platform shown in panel A into an analyzer/card system produced initially for membrane-based applications for cellular testing. (**C**) The same instrument as shown in panel B, was designed to be compatible (hardware, optics, software) with both the membrane-based and bead-based approach. Microfluidic cartridges are currently being developed to permit integration of the bead-based system with specifications consistent with the type of instrumentation shown in panels B&C. (**D**) Silicon-based microfluidic device housed in stainless steel flow cell used in benchtop configuration shown in panel A. (**E**) An injection-molded cartridge for the membrane-based system dedicated to CD4 counting was produced for instrument shown in panel B. (**F**) p-BNCs for porous bead-based testing modalities are shown in a CAD diagram and will undergo technology transfer efforts at a later stage for the instrument shown in panel C.

Likewise, porous beads, when combined with new LOC concepts, afford an opportunity to complete high-performance testing using simple instrumentation that is compatible with POC instrumentation. Shown in Figure 3 are previous instrumentation systems that have been developed in both academic research settings as well as through commercial partnerships to support the use of porous bead integrated sensing ensembles for future use in near-patient testing. The laboratory benchtop configuration used for the initial conceptual experiments here summarized consists of an array of anisotropically etched microcontainers, each holding individual porous bead-based sensing elements (Figure 3(A)). This microchip is held within a stainless steel flow cell (Figure 3(D)) with fluid delivered with external pumps and control systems. Previous translational efforts of this approach led to the evolution of a membrane-based analyzer/card system with an instrument that was designed to serve both membrane-based as well as bead-based applications (Figure 3(B,C)) and the creation of an injection-molded cartridge for the membrane-based system dedicated to CD4 counting (Figure 3(E)). The p-BNC cartridges, dedicated to serve bead-based applications, are still in the development stage with efforts dedicated to optimization of a number of microfluidic components of the cartridge format with laminate approaches, such as self-contained buffer packs, reagents, and waste reservoirs (CAD in Figure 3(F)) to attain specifications that would be suitable for integration with instruments such as ones depicted in Figure 3(B,C) and technology transfer to a manufacturing partner. Further, a series of portable and lab-based image analysis systems suitable for quantification of signal on the beads within each card are also being developed, tested and validated. Note that this instrumentation at the time of this writing is not yet commercially available. The ability to measure quickly and efficiently multiple biomarkers at the POC lends strong potential to impact clinical laboratory science after these medical microdevices move through the approval and validation stages [51,118].

## 4. Porous Bead Sensors

In contrast to a 2-dimensional planar surface of a typical microfluidic structure, high surface-to-volume ratios of porous substrates allow for enhanced sensitivities and lowers limit of detection values for delivered analytes [71,119]. Due to the sizes of biomolecule capture agents such as antibodies and limited surface area for immobilization, the capacity of binding on flat surfaces is significantly less than that for porous media [74]. The kinetics of binding of analytes to probes in highly porous media is often described as near-solution kinetics [68]. Here, reaction kinetics between a molecular analyte and an immobilized molecular capture probes occur as rates similar to those of two free molecules in solution. Further, internal transport and high binding densities characteristic of sporous substrates make these sensors suitable alternatives to current detection technologies, where rapid results are desirable for low volumes of sample containing low concentrations of target analyte.

Likewise, the p-BNC method that utilizes the porous bead as an immunosensor meets and often exceeds analytical characteristics, such as test dynamic range and limit of detection [7] of mature research or commercial instrumentation for a wide variety of analyte systems, thereby allowing dilution of the sample, if needed [24,28].

The strong analytical performance of the p-BNCs can be linked directly to the porosity and 3-dimensionality of the agarose bead capture elements. The choice of agarose is based in part on the potential for scalability, as it is derived from inexpensive sources (*i.e.*, seaweed). The same beads are already made in large quantities to support immunochromatographic applications that are dedicated to applications such as the purification of proteins. In addition to its tunable porosities (see below), this polymerized sugar matrix exhibits ultra-low nonspecific binding characteristics and the medium is index matched with water. The latter optical characteristics (unlike paper) make the material ideal not only for separation, but also as an environment (*i.e.*, a mini-cuvette) for optical detection.

Other advantages of the agarose bead sensors include (a) a capacity to be tailored so as to accommodate the specifications (such as molecular weight, size and shape) of the targeted analytes, (b) a capacity to be implemented for both two-site immunometric as well as competitive assays, (c) a capacity to be mass produced for widespread clinical purposes, (d) a capacity to be stabilized so as to withstand extreme storage conditions, (e) similar to immunochromagraphic applications, a capacity to be recycled for successive assay runs, as needed, and (f) a capacity to support fluorescence-, colorimetry-, and electrochemistry- based signal transduction [120].

### 4.1. Fibrous Network

**Figure 4.**
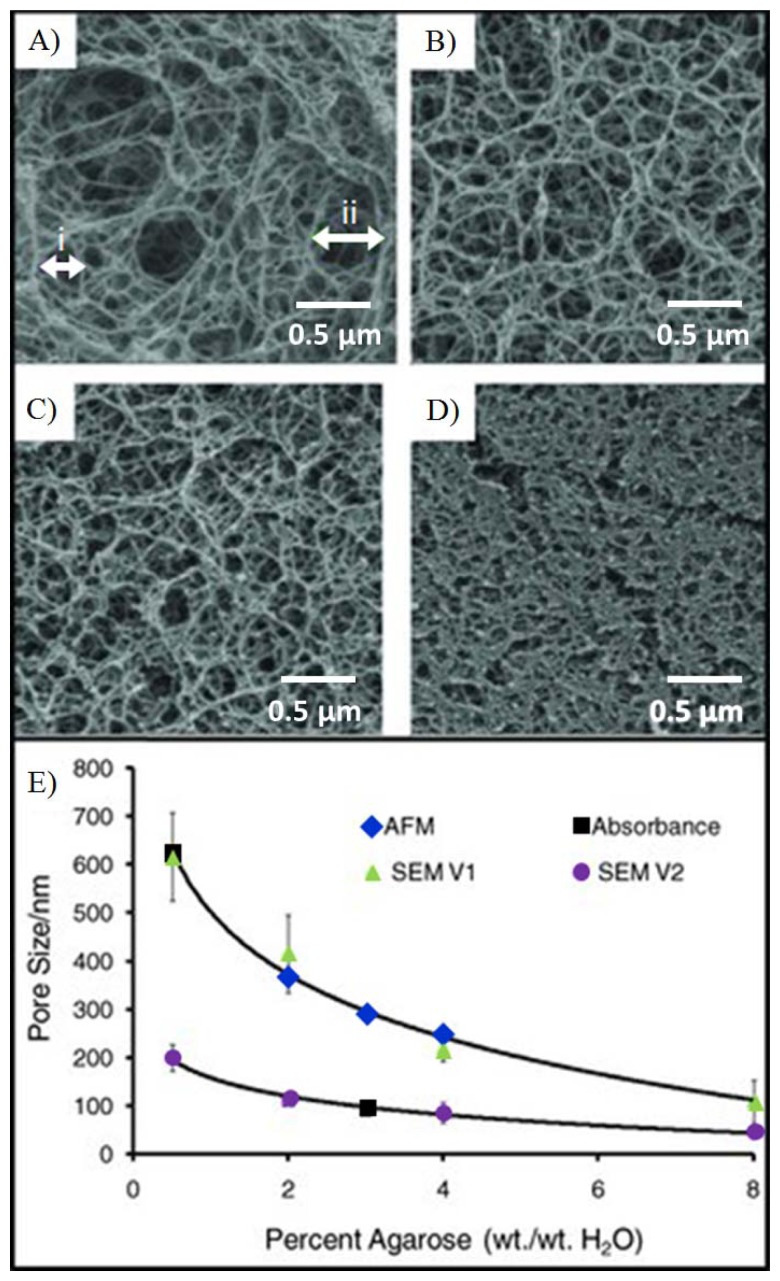
Control of weight fraction of agarose content during production serves to tune the average pore size of the beads. The SEM images, showing (**A**) 0.5%, (**B**) 2%, (**C**) 4%, and (**D**) 8% agarose by weight, reveal the decrease in pore size as the density of agarose fibers increases. Scale bars labeled (i) and (ii) in panel A demonstrate measurements used to determine average pore size of bead concentrations from 0.5–8%. (**E**) This exponentially decreasing relationship of pore size as a function of agarose percentage, calculated from different microscopy techniques [74].

Agarose beads are typically produced in bulk using standard emulsion polymerization methods [121]. By varying the agitation conditions, the gel temperature, the surfactant concentration and the feedstock concentrations, it is possible to obtain agarose beads of sizes ranging from 15 μm to more than 500 μm. Further, judicious choice of conditions can be used to isolate beads with agarose weight concentration values that vary from 0.5% to 8%. Important to note is the fact that the balance of the bead is composed of the background solvent and as such this medium serves as an ideal bridge between the solution and the solid-state support. Beads of specific diameter are further selected using sieving methods, as previous described [111]. Importantly, the control of agarose content by weight during the production of porous agarose beads enables the tunability of mass transport within the porous matrix. Figure 4 shows the inverse exponential relationship of the pore size as a function of agarose concentration. An agarose concentration of 0.5% to 8% corresponds to a porosity of 99.5% to 92%. Higher levels of porosity allow for faster mass transport of analytes to open surface areas. Because 92% to 99.5% of the bead is solvent (*i.e.*, water), the high open surface area on the agarose fibers within the porous matrix with is ideal for the capture of large biomolecules.

**Figure 5.**
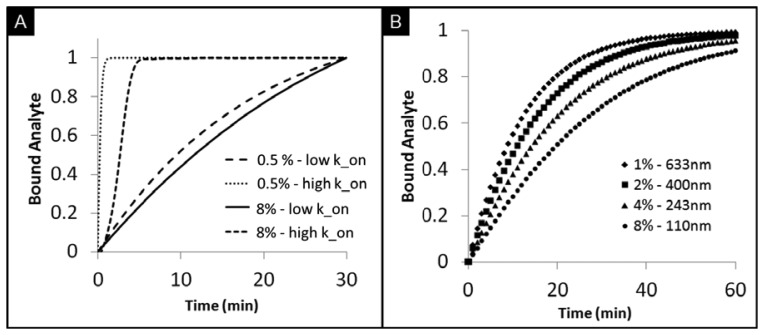
(**A**) The effect of tunability of bead porosity from 0.5% to 8% on the capture of biomarkers with low (10^4^ L/mol/s) and high (10^6^ L/mol/s) association rates (k_on) (**B**) Timecourse showing the total bound analytes on porous beads under varying agarose content.

While planar microarrays have the capacity for high-throughput testing, limited surface areas do not afford the flexibility for planar microarrays to overcome differences in timeframes and differences in concentration response curves for various analytes that need to be measured in the same setting. This limitation has resulted in slow progress in the area of protein and antibody arrays [68]. The tunability of porous beads here described, however, offers the ability to test various biomarkers through the flexibility of tunable timeframes and response curves. The customized assay performance has been demonstrated for different densities of capture probes that target CEA and IL-1β, each with different molecular weights [74]. As shown in Figure 5(A), the rates of analyte bindings widely differ depending on the binding kinetics of biomarkers. High-affinity bioanalytes bind earlier during mass transport inside the bead resulting in higher intensities at the rim of the bead. In contrast, analytes with low affinities diffuse further into the bead, which leads to lower intensities are the rim of the bead under the same timeframes. Further, the tunability of the agarose content in porous beads allows for the control of bead sensitivity and rate of binding. Figure 5(B) shows a simulation of signal development for beads with different pore sizes. With small agarose content, corresponding to larger pore sizes, the ease of transport of analytes allows for faster binding rates. A reduction of agarose content, from 8% to 0.5%, results in a decrease in the half equilibrium time from 20 min to 9 min.

Due to the small-scale dimensions of microfluidic channels, transport typically exists in the laminar flow regime. The domination of viscous forces over inertia reduces irregularities from turbulent flow [122]. As such, analytes must traverse long diffusion distances before capture. This diffusion-limited transport to the surface immobilized capture probes leads to long saturation times and low sensitivities. Gervais *et al.* has reported that the typical capture efficiency of planar microarrays, under a flow of 5 μL/min through a channel with cross-sectional area of 50 × 500 μm^2^ and a detection zone of 1mm, is only 7%. Likewise, this diffusion-limited transport leads to a 93% loss in analyte capture [123]. Further, these losses are even more significant at higher flow rates. On the other hand, computational simulations and related control experiments have shown that in the p-BNC platform the capture efficiency is 23.5% at a 400 μL/min flow rate with 4% agarose bead. To secure the same 23.5% level of capture with planar microarrays, it is necessary to slow down the flow rate and wait at least 3× longer. This extension in timeframe places some restrictions on the prospects for near-patient testing for planar microsystems that lack alternative capture methodologies.

### 4.2. Fibrous Network

**Figure 6.**
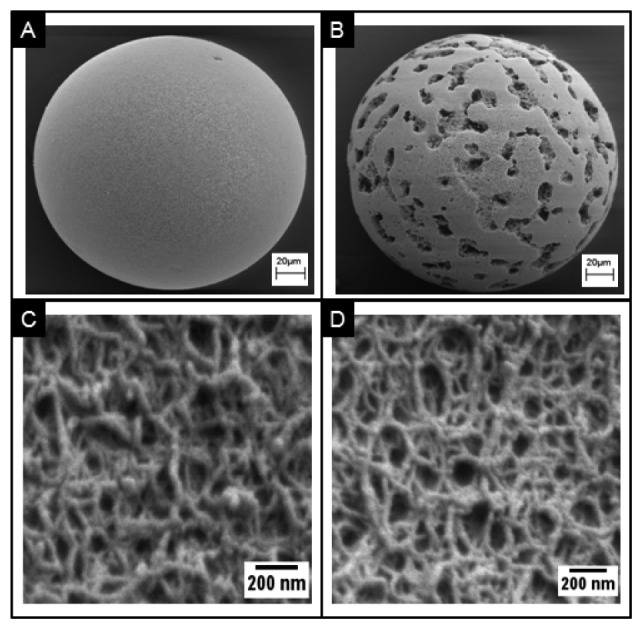
SEM images comparing porous agarose beads. (**A**) SEM images showing the surface morphology of homogenous beads containing 4% agarose and (**B**) superporous beads containing 4% agarose with ∼30 μm microcavities that allow for rapid access of fluids in the interior matrix of the bead. (**C**) Corresponding SEM images of fibrous networks for homogenous bead and (**D**) superporous bead.

The hydrophilic fibers within the porous medium allow for the functionalization of a variety of capture probes. For example, several groups have successfully demonstrated the functionalization of hydrogel for the detection of a range of analytes that include proteins, nucleotides, and cells [124–126]. Moreover, the hydrophilic surfaces of these fibers retain better protein activity over that of planar surfaces [127]. Scanning electron microscopy (Figure 6(A)) shows the surface morphology of a porous, homogenous bead created using an emulsion method [111]. While at first glance, the surface appears smooth, at higher magnification, as shown in Figure 6(C), details of the fibrous network are revealed. The densely packed nanofibers here exhibit pore sizes of approximately 100–200 nm in diameter. This range agrees with microscopy measurements within non-spherical, porous medium [128,129]. Figure 6(B) shows the surface morphology of a superporous bead. These beads, produced through double emulsion, exhibit large macropores that form interconnected tunnels within the bead. Magnification of the non-cavity regions show similar pore sizes as the homogenous case, as shown in Figure 6(D). Furthermore, because of the 3-dimensional geometry of a bead, the signals acquired from the bead are typically aggregations of thousands of layers. For example, signal on a bead is derived from a thickness that is 1,000–20,000 times larger than that from a flat monolayer on a ELISA plate [74].

Transport into the bead matrix is dependent on external flow rate and the pore size of the dense fibrous matrix [130]. Careful tuning of the pore size allows for control of the mass transport within porous beads. Thompson *et al.* developed a model in an attempt to understand the effects of analyte diffusion coefficient, flow rate, and capture probe density on the kinetics on bead surfaces [131]. Further, the combination of confocal microscopy and computational simulations helped to define the spatial and temporal distribution of bound biotinylated quantum dots on streptavidin-coated beads [132].

Prior work here has revealed the existence of internal convection within porous beads. The unique design employed by the p-BNC, consisting of individual beads in flow-through microcontainers, allows for pressure-driven convective transport of analytes within the porous matrix [117]. This pressure-driven design, which forces fluids into the porous medium, increases analyte-antibody interactions and allows for faster signal generation. When porous beads are employed in lateral flow designs, however, the convective transport within the bead matrix is limited [133,134]. Instead, the poor capture efficiency between antibody and antigen results in equilibrium saturation times of several hours [116,134]. To overcome such limitations, Bau *et al.* implemented a breathing bead methodology to expand and compress porous beads to accelerate mass transfer within such beads to increase signal intensity by a factor of ∼2.5 [135]. Within the p-BNC, the use of an intimate contact between the bead ensemble and the porous beads appears to be essential to create a pressure gradient atop the bead that can be used to facilitate the internal transport within the bead interior [136].

Further, modification of the pore size through control of agarose content in the beads allows for the increase of internal transport. In contrast to lateral flow designs, the flow-through design redirects fluids to create a high-pressure gradient that enhances internal mass transport. An adaptation of the Koreny-Carman equation shows that fluid velocity in a porous medium is proportional to applied pressure gradient and square of the pore diameter [137]. Previous studies have revealed that the internal convective transport is linearly proportional to the rate of bulk fluid delivery [138]. When the agarose concentration of the bead is increased from 0.5% to 8%, the ratio of the internal to external flow rates decreases from 1:170 to 1:3,100, equivalent to an 18-fold decrease in internal convective transport [117].

**Figure 7.**
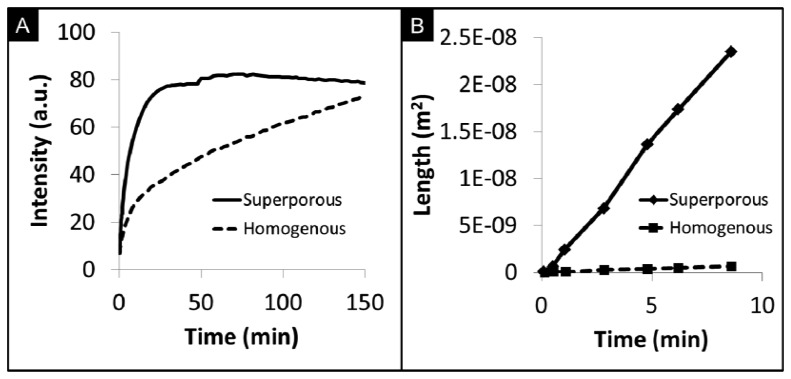
(**A**) Comparison of the capture of C-reactive protein with homogenous and superporous beads. (**B**) The diffusion rate of CRP into superporous beads is 50× higher than that for homogenous beads.

Superporous beads have shown promise to enhance the mass transport of analytes that include proteins and cells into the internal bead matrix [139–142]. In addition to the fibrous network with pore sizes between 100–800 nm, as in the case of homogenous beads, superporous beads also contain large flow cavities with diameters of 10–30 μm [74]. These cavities of micropores allow for quicker access of fluids into the bead core and reduce equilibrium times than those exhibited by homogenous beads with similar sizes of micropore. For example, Larsson *et al.* observed intra-particle fluid velocities in superporous beads to be as high as 17% of the interstitial velocity in a chromatography column [130]. Moreover, the use of superporous beads have been shown to reduce back pressure build-up in microfluidic devices [143]. Large cavities with diameters of 30 μm result in craters on the surface. Internally, these craters lead to interconnecting cavities that form long tunnels for easy access of fluids into the interior matrix (Figure 7(B)).

Figure 7(A) compares the capture of C-reactive protein (CRP) by superporous and homogenous beads. At very short timeframes, the rate of CRP capture in superporous beads is approximately 2.1× faster than homogenous beads. To reach the same fluorescence level in homogenous beads, the length of the assay would need to be extended 5-fold. Although saturation intensity is somewhat higher for homogenous beads due to the increased mass of the medium, the long duration required to reach saturation is not ideal for point-of-care time and sample volume constraints. While controlling the agarose content in homogenous beads affords higher penetration of analytes into the bead, large macropores in superporous beads allow for increased accessibility to otherwise underutilized internal binding sites [74]. As shown in Figure 7(B), analyte diffusivity in superporous beads, measured by the square penetration of signal over time, is 50× higher than the limited internal transport inside homogenous beads. The bottom line here is that the enhanced transport through availability of superpore cavities may allow for faster assay completion times while assay clinical performance would have to be compared to that of homogeneous beads.

### 4.3. High Capacity for Binding

The capacity of binding of capture probes on porous medium is two to three orders of magnitude higher than that of planar surfaces [116]. For example, the use of porous hydrogels, as substrates to immobilize antibodies, show increased capture of analytes as a result of the higher capacity for antibody immobilization [144]. As such, higher capture probe densities immobilized on beads allow for decreased limit of detections. For example, Zubstov *et al.* revealed that while the mean binding distances between antibodies in porous hydrogels is an order of magnitude higher than that of flat surfaces, fibrous 3D surfaces offer capacities that are two to three orders of magnitude higher than those available on surfaces [116]. Further, due to internal diffusion and faster binding rates, porous gel-based sensing elements exhibited higher fluorescent signals than those of their surface counterparts.

**Figure 8.**
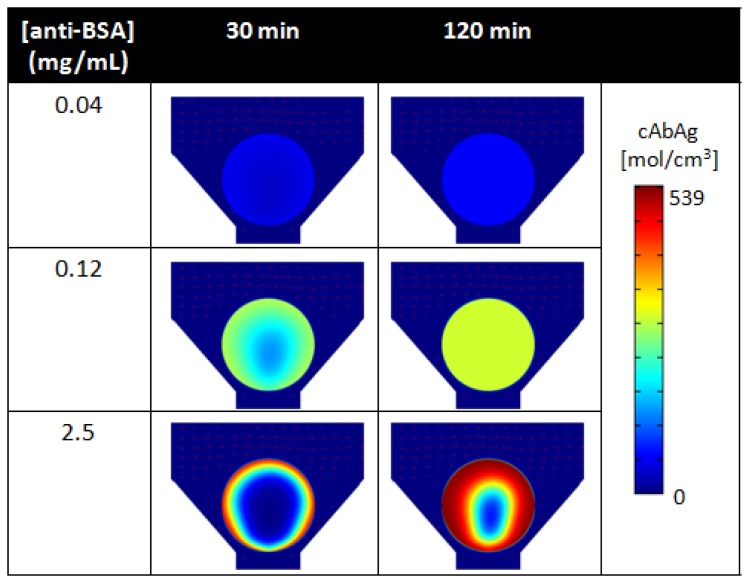
Spatial distribution of fluorescently-labeled BSA, as a function of microbead receptor concentration and time, as predicted by finite element analysis, at the diametral plane of the microbead under different binding densities.

As revealed by simulations, Figure 8 compares the spatial distribution of bound analytes within porous beads under low (0.04 mg/mL), medium (0.12 mg/mL), and high (2.5 mg/mL) binding densities of anti-BSA capture probes at 30 min and 120 min of analyte delivery. At high densities of capture probes, mass transport of free analytes to the internal core of the bead matrix is limited (Figure 8, bottom). Here, the short residence time of the free analyte results in high signal localized at the rim of the bead. However, as initial capture probes at the rim of the bead become bound, a moving boundary of bound analytes develops and penetrates radially towards the center of the bead matrix. This moving boundary continues as free analytes bind to internal capture probe sites, until the bead is completely saturated with bound analytes. In contrast, lower capture probe densities result in lower saturation intensities, but allow for faster analyte binding into the bead matrix (Figure 8, top). Here, the moving boundary permeates radially towards the center of the bead at a much faster rate than that for high capture probe densities. As shown in Figure 8, under the same timeframes, the uniform distribution of signal develops under 0.04 mg/mL of capture antibodies while a higher signal is localized at the rim of the bead under 2.5 mg/mL of capture antibodies. Because the percentage of available capture probes decrease at fast rate, signal quickly reaches equilibrium at the rim of the bead and then within the internal matrix of the bead. These results derived by simulation have also been validated through experimental studies [117].

Furthermore, the density of binding is nonlinear to the concentration of capturing antibody used for conjugation. While porous medium offers high capacities for binding, no further benefits in signal occurs as higher concentrations of probes are used for immobilization due to saturation of available sites for antibody immobilization. For example, the high, medium, low densities of capture probes shown in Figure 8, corresponding to a capture probe density of 100:4.8:1.6 ratio, result in a respective intensity ratio of 100:59:13. For superporous beads, Yang *et al.* witnessed limited signal increases for capturing antibody concentrations higher than 0.5 mg/mL [143]. While there exists a critical antibody density that does not allow for further binding, the capability for binding resulting from the high surface-to-volume ratio in the bead matrix exceeds that of planar surfaces and allows for higher sensitivities. While the total surface area for binding is limited, an increase in agarose content during the production of the beads, or higher agarose concentration, allows for more surface area for binding. As such, the interplay between porosity and capture probe concentration allows for control of bead sensitivities as well as assay response times.

## 5. Steps toward Broad-Scale Clinical Practice

Having clearly articulated the analytical performance advantages of using porous beads for biomarker capture with microstructures, it is now essential to explore the next critical steps required to use these mini-sensor ensembles in real-world clinical practice. Indeed, despite large investments in translational research programs, most bioscience research efforts remain largely decoupled from broad-scale clinical practice, both for the biomarkers as well as for the devices that measure them [7]. This general trend extends to medical microdevices and LOC systems as has been highlighted in recent perspective articles [54,145]. The typical structure associated with the device development process, whether from academia, national labs or the industrial sector, is a succession of lengthy steps that often happen in a linear, sub-optimal and disjointed manner taking considerable time and draining precious resources and momentum out of venture capital and federal funding, alike.

A case in point for the dismal rate of translation of new medical tests into real-world practice is extracted from a recent analysis by Schully *et al.* [146] of the Fiscal Year 2007 extramural grant portfolio of the National Cancer Institute (NCI), as well as cancer genetic research articles published in 2007. The group classified both funded grants and publications as follows: T0, as discovery research; T1, as research to develop a candidate health application (e.g., device or therapy); T2 as research that evaluates a candidate application and develops evidence-based recommendations; T3 as research that assesses how to integrate an evidence-based recommendation into cancer care and prevention; and T4 as research that assesses health outcomes and population impact. They found that 1.8% of the grant portfolio and 0.6% of the published literature was T2 research or beyond.

In an attempt to move the medical microdevices and porous bead sensor systems into broad-scale clinical practice, a number of clinical trials and pilot studies have been initiated. Likewise, the laboratory version of the p-BNC bead-based methodology described above along with a related membrane-based system not covered here are now involved in six clinical trials and two pilot studies, respectively, involving over 5,000 patients, including over 10 clinical sites for diseases in the areas of cardiac heart disease, ovarian cancer, prostate cancer and drugs of abuse (See Table 1) [147–149].

High-impact diseases and conditions, such as cardiovascular disease and various cancers, including ovarian cancer and prostate cancer are the targets of these p-BNC development and biomarker validation trials. The ability to use validated biomarkers in a common platform affords interesting synergies with respect to opening up new and more efficient treatment methods for management of patients [7,8]. The same platform approach is also dedicated to testing for anti-epilepsy drugs, as well as drugs of abuse, with targeted applicability in various settings, including home and the identification of drivers under the influence of drugs, at the point of arrest.

The capacity of the p-BNCs to multiplex, or simultaneously measure multiple analytes within a single assay run, is not only economically beneficial, but it also allows to test for biomarkers that collectively provide a more comprehensive look into the overall well-being of a person, while at the same time derive a more specific look to specific stages of the disease process.

Case in point, to date there are no global methods to define in an efficient way the entire cardiovascular health of patients. Today cardiac heart disease is the number one killer globally [150]. While there are many good approved biomarkers, it has not been cost effective to manage patient's care through measurement of all of these biomarkers for all patients [150]. Cost restrictions and a lack of understanding of the disease progression have limited progress in this area. With these limitations in mind, recent efforts target the development of customized cardiac chips that could evaluate the overall cardiovascular health of patients.

**Figure 9.**
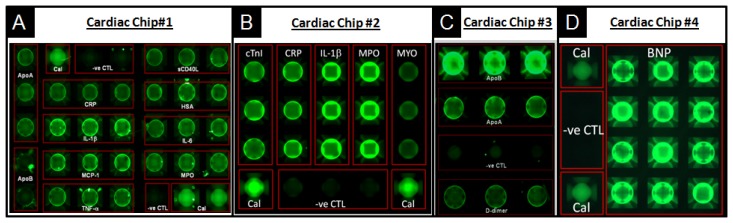
The p-BNC diagnostic applications for cardiovascular disease include (**A**) Risk for primary cardiac event chip: Apolipoprotein A1 (ApoA1), Apolipoprotein B (ApoB), C-reactive protein (CRP), Interleukin-1beta (IL-1b), Monocyte chemoattractant protein-1 (MCP-1), Tumor necrosis factor-alpha (TNF-a), Soluble CD40 ligand (sCD40L), Human serum albumin (hsa), Interleukin-6 (IL-6) and Myeloperoxidase (MPO); (**B**) Expanded AMI diagnosis chip: Cardiac troponin I (cTnI), Myoglobin (CRP), IL-1β, MPO and MYO; (**C**) Risk for secondary cardiac events chip: ApoA1, ApoB and D-dimer; and (**D**) Congestive heart failure chip: Brain natriuretic peptide (BNP)

For example, biomarkers for all aspects of care regarding cardiovascular disease, including diagnosis of acute myocardial infarction, prognosis or risk for secondary cardiac events, monitoring, risk stratification and guidance for therapeutic interventions of cardiac patients, were developed as multiplexed panels [150]. Figure 9 details the use of porous agarose beads, as supported in the p-BNC in this capacity. Here, four panels of multiplex biomarkers target different cardiovascular diseases that include the risk for primary cardiac event, expanded acute myocardial infarction (AMI) diagnosis, risk for secondary cardiac events, and congestive heart failure. Panel sizes and bead sensors are easily swapped to serve the needs of the diagnostic application. Noted for each array is the redundancy of bead sensors per analyte, which contributes to accurate and precise measurements. Also, noted here is the presence of calibrator beads (Cal), which serve for the baseline calibration of the p-BNC system, and negative control (-ve CTL) beads, coupled to antibodies irrelevant to any of the analytes targeted in each test, which serve as indicators of the specificity of the antigen-antibody reactions that take place within the lab card.

Key to future successful implementation of these tests is the fact that they meet the analytical performance requirements, as dictated by the pathophysiological levels of the various biomarkers for healthy *vs.* diseased. Likewise, in order for these chip-based tests to have clinical relevance, they must not only respond on a timeframe consistent with near-patient usage, but they must meet or exceed the analytical, and, thereby, clinical performance of the gold standards or reference methods, that are for the most part limited to the laboratory setting (see Table 2). With this in mind, a significant amount of effort has now been devoted to the determination of the analytical performance of key biomarkers using the porous bead sensor systems. These measurements are completed using human clinical samples and thus move above and beyond the common starting place of purified antigens within buffer solutions.

With this real-world clinical context in mind, the limits of detection (LODs) for the two-site immunometric and competitive assays have been established as reported in Table 2. Here both practical and theoretical methods for determination of LOD values have been employed [151–153]. The former method was used for some of the initial work and the latter for the majority of the more recent activities. For the theoretical LOD values, a 4- or 5-parameter logistic curve fit was applied to the dose response curve and then the intersection with the intensity level three standard deviations above the zero calibrator mean of three trials was established to yield the concentration value that defines the LOD. In cases where no ultra-low concentration standards were run, the practical method was used. Here the LOD was established as the lowest concentration of antigen yielding an average bead signal that lies at three standard deviations above (two-type immunometric) or below (competitive) the mean value recorded for the zero analyte calibrator.

From Table 2 it can be seen that the p-BNC tests for drugs of abuse exhibit exceptional assay performance characteristics with limits of detection (LODs) comparable to the laboratory-confined reference method of LC-MS/MS and vastly superior (significantly lower) than their immunochromatographic (ICS) counterpart tests. Similar to LC-MS/MS and unlike ICS tests, which are qualitative (Yes/No) type of tests, p-BNC-based drug tests are fully quantitative. Furthermore, unlike LC-MS/MS which requires tedious sample processing and is limited to testing for one drug at a time, p-BNC tests once in their final format, will be amenable to the point of need, require no extensive sample processing and offer the capacity to multiplex, or test more than one analyte (drug) concurrently, using microliters of sample.

## 6. Current Bead-Based Analyzers

Table 3 showcases current bead-based analyzers and the timeframes to complete the test. Given the timeframes to perform an analysis, most analyzers based on solid support bead sensors are confined to testing in the clinical laboratory. For example, the Luminex xMap system, which employs a suspension array technology to detect multiple analytes simultaneously, has a timeframe for detection that is in the range of two hours to overnight. It is apparent that the timeframes offered would not be ideal for point-of-care testing. As such, an increase in the sensitivity of bead-based approaches would lend to faster analysis times that are suitable for point-of-care testing.

The combination of porous support beads and tailored microstructures with active transport features allows for enhanced mass transport of analytes. Short analysis timeframes of these highly sensitive sensing elements reduce analysis times achieving what would be consistent with the point-of-care.

## 7. Scalability

As mentioned above, in order for the medical microdevices area to reach their full potential, it will be necessary for these mini-test ensembles and their associated measurement platforms to be fully validated with results that exhibit strong performance characteristics that rival those achieved in traditional laboratory settings [7]. Further, it will be necessary that these devices show scalability and performance that exceeds existing laboratory capabilities. To be scalable, enhanced performance must be achieved with reduced cost and increased efficiencies.

The pathway to scalability would be expected to occur with modular assay formats that encompass major diagnostic classes and with the establishment of standard testing procedures [7]. It is in this capacity that the microelectronics industry should be considered to be a key model for the diagnostics industry to follow. While initial discoveries in the electronics area, such as the vacuum tube, offered basic logical operations, large mainframes and electrical component failures limited the widespread adoption. However, with the advent of the solid-state three-point transistor and photolithographic processing that brought standards and scalability, the microelectronics industry pushed the limits of its capabilities as components became smaller and more powerful. Furthermore, for this now dominant industry, Moore's law serves as a goal to produce increased computational power at reduced cost.

Unfortunately, today healthcare costs are increasing at an alarming rate with only modest increases in the quality of patient care [154]. Today over 60%–70% of medical decisions are made with consultation of clinical testing results, yet less than 5% of total costs in this space are associated with the testing results [155]. Likewise, it is clear that there is good value in clinical testing and information content here derived will play an important role for the future management of healthcare and wellness.

**Figure 10.**
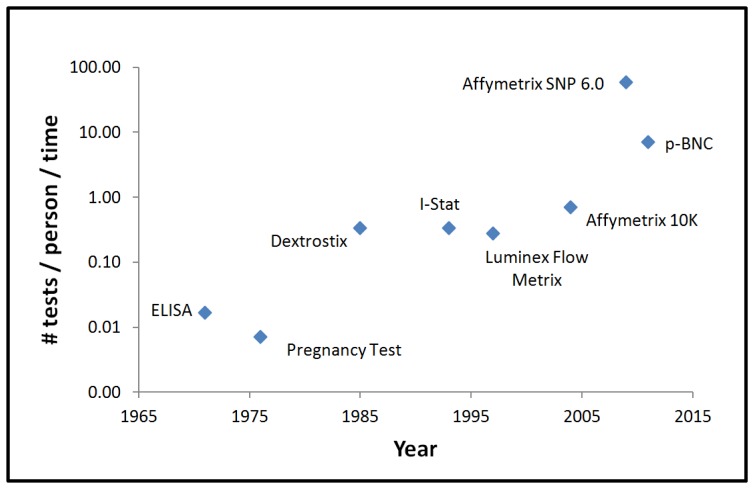
Graph over time showing the increase in Q, the number of tests performed per person per time, for various representative diagnostic approaches.

It might be expected, therefore, that the development of scalable high-performance diagnostic platforms alongside Moore's law-like goals could have potential to lead to transformative changes in healthcare. Figure 10 provides a representative list of diagnostic instruments and shows how the number of tests that are performed per session have evolved with time. In assembling this information, the “Q” quotient is used, as this value designates the number of tests that are performed per person per time [75]. With this quantity in mind, the focus is placed directly on the information content as needed to impact clinical decision making for the individual patient. In this capacity, the time value here designated includes transit, preparation, and analysis periods. From this graph, it is quite interesting to note that bead-based systems and chip-based approaches are now providing the highest level of information extraction to date. It is also interesting to note that, similar to the field of microelectronics, there is an evolving trend with time to secure more biomarker-derived information.

For the purpose of this graph, it is assumed that all the information content obtained from microarray discovery data is used in the clinical decision making process. However, more typically only a small number of sequences contribute to the differential disease diagnosis and thus these specific signatures serve as a small portion of the acquired information. In the future it might be expected that as these devices move closer to widespread clinical practice, a stronger focus on key information from the selected sequences derived from more efficient capture methods will evolve. Integrated LOC systems with improved efficiencies in sample processing are expected to play key roles in extracting the key information that is required to manage patient care in cost effective and efficient ways moving forward.

## 8. Conclusions

In many ways, the convergence of microfluidics, biomarker validation, and porous bead ensembles serve to overcome some of the significant challenges that the medical microdevices have, to date, faced with respect to scalability and performance. When combined with new concepts for noninvasive sampling [156], there is now strong potential to move these sensor modalities into broad-scale clinical practice. Before this is possible, it will be necessary to complete more thorough clinical testing and validation. The ability to complete multiplexed testing on porous beads that can be customized for response time allows for multiple biomarkers to be measured within one environment in ways that would not readily be achieved with more traditional planar approaches. Further, the prospect for using the same diagnostic core in the discovery, clinical validation and broad-scale testing serves to establish a promising pathway that has potential to increase the pace of both new device and new biomarker approvals [7].

Further, the power of scalability through reduced time per test could lead the way to a new generation of diagnostic and detection tools for health care applications. These devices, with the ability to quickly provide robust, affordable, and accurate results, could potentially diagnose diseases at early stages, monitor health risks, manage illness and provide appropriate treatment options [37]. The ability to detect disease early on can improve the quality of the patient life, influence life style changes, and reduce overall treatment costs. As such, these advanced medical microdevices may serve as the enabling technology base that can help to usher in long awaited transition from reactive to preventative medicine [54].

## Figures and Tables

**Table 1.** The bead-based p-BNC is involved in six clinical trials through several major sponsors to target a number of diseases though the validation of a number of biomarkers.

Study	Sponsor	Area	# Of Subjects	Clinical Site	Biomarkers
Development of A Lab-on-a-Chip System for Saliva-Based Diagnostics	National Institute of Dental and Craniofacial Research (NIDCR)	Cardiac Disease	1,050 patients	Baylor College of Medicine	15 proteins
Advanced Bio-Nano-Chips for Saliva-Based Drug Tests at the Point of Arrest	Home Office-Center of Applied Science and Technology (HO-CAST)	Drugs of Abuse	340 participants	Baylor College of Medicine	12 drugs
Texas Cancer Diagnostics Pipeline Consortium	Cancer Prevention Research Institute of Texas (CPRIT)	Ovarian Cancer	2,660 patients	MD Anderson Cancer Clinic	4 proteins
	Cancer Prevention Research Institute of Texas (CPRIT)	Prostate Cancer	1,100 patients	UT Health Science Center-San Antonio	3 proteins
Pilot and Prospective Studies for the Development of the Trauma Chip	Texas Emerging Technology Fund	Acute Kidney Failure	120 patients	UT Health Science Center-Houston	5 proteins
Development of p-BNCs for the Monitoring of Anti-Epilepsy Drugs Levels in Saliva	John S. Dunn Foundation	Epilepsy	100 patients	UT Health Science Center-Houston	3 proteins

**Table 2.** Initial specifications obtained with laboratory-based p-BNC porous bead-based approach: List of developed biomarker assays, targeted use, and device performance characteristics in the context of real-world clinical testing.

**Biomarker**	**Clinical Use**	**Range a (ng/mL)**	**LOD a (ng/mL)**	**Method**
C-reactive protein	AMI, Risk Assessment	0.1–10,000	0.1	Theoretical
Soluble CD40 ligand	Cardiac Risk Assessment	0.1–1,000	0.1	Practical
Monocyte chemoattractant protein-1	Cardiac Risk Assessment	0.001–20	0.001	Practical
Myeloperoxidase	Cardiac Risk Assessment	0.05–500	0.05	Practical
Myeloperoxidase (multiplexed)	Cardiac Risk Assessment	1.2–500	1.2	Theoretical
Interleukin-1beta	Cardiac Risk Assessment	0.001–1	0.001	Practical
Interleukin-6	Cardiac Risk Assessment	0.001–1	0.001	Practical
Tumor necrosis factor-alpha	Cardiac Risk Assessment	0.01–10	0.01	Practical
Cardiac troponin I	AMI Diagnosis	0.05–50	0.05	Theoretical
Myoglobin	AMI Diagnosis	0.1–1,000	0.1	Theoretical
CK-MB	AMI Diagnosis	1.7–50	1.7	Theoretical
Apolipoprotein A1	Risk for recurrence/Prognosis	1–1,000	1	Practical
Apolipoprotein B	Risk for recurrence/Prognosis	1–1,000	1	Practical
Brain natriuretic peptide	Congestive Heart Failure	0.05–10	0.05	Theoretical
N-Terminal proBNP	Congestive Heart Failure	0.1–500	0.1	Theoretical
Human serum albumin	Cardiac Risk Assessment	1–1,000	1	Practical
Transferrin	Blood contamination in saliva	0.05–10,000 b	0.05 b	Theoretical
Carcinoembryonic antigen	Ovarian Cancer	0.1–100	0.02	Theoretical
Cancer antigen 125	Ovarian Cancer	1–400 c	1 c	Theoretical
Human ep growth fact Rec. 2-neu	Ovarian Cancer	0–60	0.27	Theoretical
Prostate-specific antigen	Prostate Cancer	0.1–100	0.1	Theoretical
Free prostate-specific antigen	Prostate Cancer	0.1–100	0.1	Theoretical
Complexed prostate-specific antigen	Prostate Cancer	0.63–100	0.63	Theoretical
Cocaine	Road Side Drug Testing	1.3–10,000	1.3	Practical
Diazepam	Road Side Drug Testing	0.14–1,000	0.14	Practical
Tetrahydrocannabinol	Road Side Drug Testing	0.22–10,000	0.22	Practical
D-Amphetamine	Road Side Drug Testing	0.22–1,000	0.22	Practical
Methamphetamine	Road Side Drug Testing	10–8,000	1	Practical
Oxazepam	Road Side Drug Testing	1.6–100,000	1.6	Theoretical
Nordiazepam	Road Side Drug Testing	0.72–100,000	0.72	Theoretical
Temazepam	Road Side Drug Testing	1.1–100,000	1.1	Theoretical
Morphine	Road Side Drug Testing	0.46–1,000	0.46	Theoretical
Methadone	Road Side Drug Testing	1.02–10,000	1.02	Theoretical
MDA	Road Side Drug Testing	7.1–1,000	7.1	Theoretical
MDMA	Road Side Drug Testing	0.41–1,000	0.41	Theoretical

a all units are ng/mL unless otherwise specified.

b units are expressed here as μg/mL.

c units are expressed here as U/mL.

**Table 3.** List of bead-based instruments, number of different bead sensors for multiplexing, and total time to complete a test.

**Instrument**	**Source**	**Total Tests**	**Time**	**Setting**
xMap	Luminex	500	390 min	Laboratory
BD FACSArray	BD	35	35 min	Laboratory
AtheNA Multi-Lyte	Alere	26	60 min	Laboratory
VeraCode BeadXpress	Illumina	48	30 min	Laboratory
Liaison	Diasorin	144	60 min	Laboratory
p-BNC *	McDevitt Lab	100	20 min	Lab & POC

* The analyzer and biochips associated with the p-BNC approach for porous beads are still in development and are not yet commercially available. Specifications are being developed for future technology transfer through commercial partnerships. Characteristics listed in the table are projected for ultimate use.

## References

[1] Hanash S.M., Pitteri S.J., Faca V.M. (2008). Mining the plasma proteome for cancer biomarkers. Nature.

[2] Hunter D.J., Khoury M.J., Drazen J.M. (2008). Letting the genome out of the bottle—Will we get our wish?. N. Engl. J. Med..

[3] Anderson N.L. (2010). The clinical plasma proteome: A survey of clinical assays for proteins in plasma and serum. Clin. Chem..

[4] Anderson N.L., Anderson N.G. (2002). The human plasma proteome. Mol. Cell. Proteomics.

[5] Vitzthum F., Behrens F., Anderson N.L., Shaw J.H. (2005). Proteomics: From basic research to diagnostic application. A review of requirements & needs. J. Proteome Res..

[6] Paulovich A.G., Whiteaker J.R., Hoofnagle A.N., Wang P. (2008). The interface between biomarker discovery and clinical validation: The tar pit of the protein biomarker pipeline. Proteomics Clin. Appl..

[7] Jokerst J.V., Jacobson J.W., Bhagwandin B.D., Floriano P.N., Christodoulides N., McDevitt J.T. (2010). Programmable nano-bio-chip sensors: Analytical meets clinical. Anal. Chem..

[8] Floriano P.N., Christodoulides N., Miller C.S., Ebersole J.L., Spertus J., Rose B.G., Kinane D.F., Novak M.J., Steinhubl S., Acosta S. (2009). Use of saliva-based nano-biochip tests for acute myocardial infarction at the point of care: A feasibility study. Clin. Chem..

[9] Yager P., Edwards T., Fu E., Helton K., Nelson K., Tam M.R., Weigl B.H. (2006). Microfluidic diagnostic technologies for global public health. Nature.

[10] Soper S.A., Brown K., Ellington A., Frazier B., Garcia-Manero G., Gau V., Gutman S.I., Hayes D.F., Korte B., Landers J.L. (2006). Point-of-care biosensor systems for cancer diagnostics/prognostics. Biosen. Bioelectron..

[11] Myers F.B., Lee L.P. (2008). Innovations in optical microfluidic technologies for point-of-care diagnostics. Lab Chip.

[12] Ouellette A.L., Li J.J., Cooper D.E., Ricco A.J., Kovacs G.T. (2009). Evolving point-of-care diagnostics using up-converting phosphor bioanalytical systems. Anal. Chem..

[13] Osterfeld S.J., Yu H., Gaster R.S., Caramuta S., Xu L., Han S.J., Hall D.A., Wilson R.J., Sun S., White R.L. (2008). Multiplex protein assays based on real-time magnetic nanotag sensing. Proc. Nat. Acad. Sci. USA.

[14] Lewandrowski K. (2009). Point-of-care testing: An overview and a look to the future (circa 2009, United States). Clin. Lab Med..

[15] Melanson S.E.F. (2011). Literature review on point-of-care testing (August 2009–December 2010). Point of Care.

[16] Durner J. (2010). Clinical chemistry: Challenges for analytical chemistry and the nanosciences from medicine. Angew. Chem. Int. Ed..

[17] Gervais L., de Rooij N., Delamarche E. (2011). Microfluidic chips for point-of-care immunodiagnostics. Advan. Mater..

[18] Warsinke A. (2009). Point-of-care testing of proteins. Anal. Bioanal. Chem..

[19] Gubala V., Harris L.F., Ricco A.J., Tan M.X., Williams D.E. (2011). Point of care diagnostics: Status and future. Anal. Chem..

[20] Thorsen T., Maerkl S.J., Quake S.R. (2002). Microfluidic large-scale integration. Science.

[21] Mukhopadhyay R. (2009). Microfluidics: On the slope of enlightenment. Anal. Chem..

[22] Haeberle S., Zengerle R. (2007). Microfluidic platforms for lab-on-a-chip applications. Lab Chip.

[23] Schulte T.H., Bardell R.L., Weigl B.H. (2002). Microfluidic technologies in clinical diagnostics. Clin. Chim. Acta.

[24] Luppa P.B., Müller C., Schlichtiger A., Schlebusch H. (2011). Point-of-care testing (poct): Current techniques and future perspectives. TrAC Trend. Anal. Chem..

[25] Wu P., Castner D.G., Grainger D.W. (2008). Diagnostic devices as biomaterials: A review of nucleic acid and protein microarray surface performance issues. J. Biomater. Sci.-Poly. Ed..

[26] Pabbaraju K., Tokaryk K.L., Wong S., Fox J.D. (2008). Comparison of the luminex xtag respiratory viral panel with in-house nucleic acid amplification tests for diagnosis of respiratory virus infections. J. Clin. Microbiol..

[27] Sista R., Hua Z., Thwar P., Sudarsan A., Srinivasan V., Eckhardt A., Pollack M., Pamula V. (2008). Development of a digital microfluidic platform for point of care testing. Lab Chip.

[28] Stewart D.S., Reineke K.F., Tortorello M.L. (2002). Comparison of assurance gold salmonella eia, bax for screening/salmonella, and gene-trak salmonella dlp rapid assays for detection of salmonella in alfalfa sprouts and sprout irrigation water. J. AOAC Int..

[29] Bourbeau P.P., Heiter B.J., Figdore M. (1997). Use of gen-probe accuprobe group b streptococcus test to detect group b streptococci in broth cultures of vaginal-anorectal specimens from pregnant women: Comparison with traditional culture method. J. Clin. Microbiol..

[30] Vickers I., O'Flanagan D., Cafferkey M., Humphreys H. (2011). Multiplex pcr to determine streptococcus pneumoniae serotypes causing otitis media in the republic of ireland with further characterisation of antimicrobial susceptibilities and genotypes. Eur. J. Clin. Microbiol. Infect. D.

[31] Keiler K.C., Shapiro L., Williams K.P. (2000). Tmrnas that encode proteolysis-inducing tags are found in all known bacterial genomes: A two-piece tmrna functions in caulobacter. Proc. Nat. Acad. Sci. USA.

[32] McGuinness S., McCabe E., O'Regan E., Dolan A., Duffy G., Burgess C., Fanning S., Barry T., O'Grady J. (2009). Development and validation of a rapid real-time pcr based method for the specific detection of salmonella on fresh meat. Meat Sci..

[33] O'Grady J., Lacey K., Glynn B., Smith T.J., Barry T., Maher M. (2009). Tmrna—A novel high-copy-number rna diagnostic target—Its application for staphylococcus aureus detection using real-time nasba. FEMS Microbiol. Lett..

[34] Dimov I.K., Garcia-Cordero J.L., O'Grady J., Poulsen C.R., Viguier C., Kent L., Daly P., Lincoln B., Maher M., O'Kennedy R. (2008). Integrated microfluidic tmrna purification and real-time nasba device for molecular diagnostics. Lab Chip.

[35] Toner M., Irimia D. (2005). Blood-on-a-chip. Annu. Rev. Biomed. Eng..

[36] Chin C.D., Linder V., Sia S.K. (2007). Lab-on-a-chip devices for global health: Past studies and future opportunities. Lab Chip.

[37] Weigl B., Domingo G., Labarre P., Gerlach J. (2008). Towards non- and minimally instrumented, microfluidics-based diagnostic devices. Lab Chip.

[38] Martinez A.W., Phillips S.T., Carrilho E., Thomas S.W., Sindi H., Whitesides G.M. (2008). Simple telemedicine for developing regions: Camera phones and paper-based microfluidic devices for real-time, off-site diagnosis. Anal. Chem..

[39] Yager P., Domingo G.J., Gerdes J. (2008). Point-of-care diagnostics for global health. Annu. Rev. Biomed. Eng..

[40] Lee W.G., Kim Y.G., Chung B.G., Demirci U., Khademhosseini A. (2010). Nano/microfluidics for diagnosis of infectious diseases in developing countries. Adv. Drug Deliv. Rev..

[41] Fu E., Yager P., Floriano P.N., Christodoulides N., McDevitt J.T. (2011). Perspective on diagnostics for global health. IEEE Pulse.

[42] Sato K., Hibara A., Tokeshi M., Hisamoto H., Kitamori T. (2003). Integration of chemical and biochemical analysis systems into a glass microchip. Anal. Sci..

[43] Vilkner T., Janasek D., Manz A. (2004). Micro total analysis systems. Recent developments. Anal. Chem..

[44] Sorger P.K. (2008). Microfluidics closes in on point-of-care assays. Nat. Biotech..

[45] Goluch E.D., Nam J.M., Georganopoulou D.G., Chiesl T.N., Shaikh K.A., Ryu K.S., Barron A.E., Mirkin C.A., Liu C. (2006). A bio-barcode assay for on-chip attomolar-sensitivity protein detection. Lab Chip.

[46] Qin L., Vermesh O., Shi Q., Heath J.R. (2009). Self-powered microfluidic chips for multiplexed protein assays from whole blood. Lab Chip.

[47] Sia S.K., Linder V., Parviz B.A., Siegel A., Whitesides G.M. (2004). An integrated approach to a portable and low-cost immunoassay for resource-poor settings. Angew. Chem. Int. Ed. Eng..

[48] Srivastava N., Brennan J.S., Renzi R.F., Wu M., Branda S.S., Singh A.K., Herr A.E. (2009). Fully integrated microfluidic platform enabling automated phosphoprofiling of macrophage response. Anal. Chem..

[49] Kim J.S., Anderson G.P., Erickson J.S., Golden J.P., Nasir M., Ligler F.S. (2009). Multiplexed detection of bacteria and toxins using a microflow cytometer. Anal. Chem..

[50] Madou M., Zoval J., Jia G., Kido H., Kim J., Kim N. (2006). Lab on a CD. Annu. Rev. Biomed. Eng..

[51] Walt D.R. (2005). Chemistry. Miniature analytical methods for medical diagnostics. Science.

[52] Cheng X., Gupta A., Chen C., Tompkins R.G., Rodriguez W., Toner M. (2009). Enhancing the performance of a point-of-care cd4+ t-cell counting microchip through monocyte depletion for hiv/aids diagnostics. Lab Chip.

[53] Maheswaran S., Sequist L.V., Nagrath S., Ulkus L., Brannigan B., Collura C.V., Inserra E., Diederichs S., Iafrate A.J., Bell D.W. (2008). Detection of mutations in EGFR in circulating lung-cancer cells. N. Eng. J. Med..

[54] Whitesides G.M. (2006). The origins and the future of microfluidics. Nature.

[55] Hartmann M., Roeraade J., Stoll D., Templin M., Joos T. (2009). Protein microarrays for diagnostic assays. Anal. Bioanal. Chem..

[56] Hall D.A., Ptacek J., Snyder M. (2007). Protein microarray technology. Mech. Age. Dev..

[57] Gresham D., Dunham M.J., Botstein D. (2008). Comparing whole genomes using DNA microarrays. Nat. Rev. Genet..

[58] Tomlinson I.M., Holt L.J. (2001). Protein profiling comes of age. Genome Biol..

[59] Venkatasubbarao S. (2004). Microarrays—Status and prospects. Trends Biotechnol..

[60] Rifai N., Gillette M.A., Carr S.A. (2006). Protein biomarker discovery and validation: The long and uncertain path to clinical utility. Nat. Biotech..

[61] Lim C.T., Zhang Y. (2007). Bead-based microfluidic immunoassays: The next generation. Biosens. Bioelectron..

[62] Verpoorte E. (2003). Beads and chips: New recipes for analysis. Lab Chip.

[63] Bangs L.B. (1996). New Developments in Particle-Based Immunoassays: Introduction. Pure Appl. Chem..

[64] Meza M.B. (2000). Bead-based hts applications in drug discovery. Drug Discov. Today..

[65] Vignali D.A.A. (2000). Multiplexed particle-based flow cytometric assays. J. Immunol. Meth..

[66] Brodsky A.S., Johnston A.P., Trau M., Silver P.A. (2003). Analysis of RNA-protein interactions by flow cytometry. Curr. Opin. Mol. Ther..

[67] Kawaguchi H. (2000). Functional polymer microspheres. Prog. Polym. Sci..

[68] Derveaux S., Stubbe B.G., Braeckmans K., Roelant C., Sato K., Demeester J., De Smedt S.C. (2008). Synergism between particle-based multiplexing and microfluidics technologies may bring diagnostics closer to the patient. Anal. Bioanal. Chem..

[69] Wilson R., Cossins A.R., Spiller D.G. (2006). Encoded microcarriers for high-throughput multiplexed detection. Angew. Chem. Int. Ed. Eng..

[70] Kim J., Heo J., Crooks R.M. (2006). Hybridization of DNA to bead-immobilized probes confined within a microfluidic channel. Langmuir.

[71] Sato K., Tokeshi M., Odake T., Kimura H., Ooi T., Nakao M., Kitamori T. (2000). Integration of an immunosorbent assay system: Analysis of secretory human immunoglobulin a on polystyrene beads in a microchip. Anal. Chem..

[72] Zammatteo N., Alexandre I., Ernest I., Le L., Brancart F., Remacle J. (1997). Comparison between microwell and bead supports for the detection of human cytomegalovirus amplicons by sandwich hybridization. Anal. Biochem..

[73] Walsh M.K., Wang X., Weimer B.C. (2001). Optimizing the immobilization of single-stranded DNA onto glass beads. J. Biochem. Biophys. Meth..

[74] Jokerst J.V., Chou J., Camp J.P., Wong J., Lennart A., Pollard A.A., Floriano P.N., Christodoulides N., Simmons G.W., Zhou Y. (2011). Location of biomarkers and reagents within agarose beads of a programmable bio-nano-chip. Small.

[75] Jokerst J.V., McDevitt J.T. (2010). Programmable nano-bio-chips: Multifunctional clinical tools for use at the point-of-care. Nanomedicine.

[76] Delehanty J.B., Ligler F.S. (2002). A microarray immunoassay for simultaneous detection of proteins and bacteria. Anal. Chem..

[77] Konry T., Hayman R.B., Walt D.R. (2009). Microsphere-based rolling circle amplification microarray for the detection of DNA and proteins in a single assay. Anal. Chem..

[78] Situma C., Hashimoto M., Soper S.A. (2006). Merging microfluidics with microarray-based bioassays. Biomol. Eng..

[79] Ng J.K., Selamat E.S., Liu W.T. (2008). A spatially addressable bead-based biosensor for simple and rapid DNA detection. Biosens. Bioelectron..

[80] Zhao Y., Zhao X., Pei X., Hu J., Zhao W., Chen B., Gu Z. (2009). Multiplex detection of tumor markers with photonic suspension array. Anal. Chim. Acta.

[81] Walt D.R. (2010). Fibre optic microarrays. Chem. Soc. Rev..

[82] Walt D.R. (2010). Bead-based optical fiber arrays for artificial olfaction. Curr. Opin. Chem. Biol..

[83] Walt D.R. (2000). Techview: Molecular biology. Bead-based fiber-optic arrays. Science.

[84] Nolan J.P., Lauer S., Prossnitz E.R., Sklar L.A. (1999). Flow cytometry: A versatile tool for all phases of drug discovery. Drug Discov. Today.

[85] Nolan J.P., Sklar L.A. (2002). Suspension array technology: Evolution of the flat-array paradigm. Trends Biotechnol..

[86] Kuckuck F.W., Edwards B.S., Sklar L.A. (2001). High throughput flow cytometry. Cytometry.

[87] Nolan J.P., Mandy F.F. (2001). Suspension array technology: New tools for gene and protein analysis. Cell. Mol. Biol..

[88] Diaz M.R., Fell J.W. (2005). Use of a suspension array for rapid identification of the varieties and genotypes of the cryptococcus neoformans species complex. J. Clin. Microbiol..

[89] Khan I.H., Krishnan V.V., Ziman M., Janatpour K., Wun T., Luciw P.A., Tuscano J. (2009). A comparison of multiplex suspension array large-panel kits for profiling cytokines and chemokines in rheumatoid arthritis patients. Cytometry Part B.

[90] Dunbar S.A. (2006). Applications of luminex xmap technology for rapid, high-throughput multiplexed nucleic acid detection. Clin. Chim. Acta.

[91] Nolan J.P., Yang L., van der Heyde H.C. (2001). Reagents and Instruments for Multiplexed Analysis Using Microparticles. Current Protocols in Cytometry.

[92] Fulton J.R., McDade R.L., Smith P.L., Kienker L.J., Kettman J.R. (1997). Advanced multiplexed analysis with the flowmetrixtm system. Clin. Chem..

[93] Taylor J.D., Briley D., Nguyen Q., Long K., Iannone M.A., Li M.S., Ye F., Afshari A., Lai E., Wagner M. (2001). Flow cytometric platform for high-throughput single nucleotide polymorphism analysis. Biotechniques.

[94] Oliver K.G., Kettman J.R., Fulton R.J. (1998). Multiplexed analysis of human cytokines by use of the flowmetrix system. Clin. Chem..

[95] Prabhakar U., Eirikis E., Miller B.E., Davis H.M. (2005). Multiplexed cytokine sandwich immunoassays: Clinical applications. Methods Mol. Med..

[96] Prabhakar U., Eirikis E., Davis H.M. (2002). Simultaneous quantification of proinflammatory cytokines in human plasma using the labmap assay. J. Immunol. Method..

[97] Dunbar S.A., Vander Zee C.A., Oliver K.G., Karem K.L., Jacobson J.W. (2003). Quantitative, multiplexed detection of bacterial pathogens: DNA and protein applications of the luminex labmap system. J. Immunol. Method..

[98] Dunbar S.A., Jacobson J.W. (2005). Rapid screening for 31 mutations and polymorphisms in the cystic fibrosis transmembrane conductance regulator gene by lminex xmap suspension array. Methods Mol. Med..

[99] Yan X., Zhong W., Tang A., Schielke E.G., Hang W., Nolan J.P. (2005). Multiplexed flow cytometric immunoassay for influenza virus detection and differentiation. Anal. Chem..

[100] Dasso J., Lee J., Bach H., Mage R.G. (2002). A comparison of elisa and flow microsphere-based assays for quantification of immunoglobulins. J. Immunol. Method..

[101] Morgan E., Varro R., Sepulveda H., Ember J.A., Apgar J., Wilson J., Lowe L., Chen R., Shivraj L., Agadir A. (2004). Cytometric bead array: A multiplexed assay platform with applications in various areas of biology. Clin. Immunol..

[102] Gorris H.H., Blicharz T.M., Walt D.R. (2007). Optical-fiber bundles. FEBS J..

[103] Stitzel S.E., Aernecke M.J., Walt D.R. (2011). Artificial noses. Annu. Rev. Biomed. Eng..

[104] Walt D.R. (2006). Fiber optic array biosensors. Biotechniques.

[105] Stevens T.W., Iwaki Y. (2008). Introduction to bioarray solutions' beadchip technology. ASHI Quart..

[106] Thompson J.A. (2011). Microbead-Based Biosensing in Microfluidic Devices. Ph.D. Thesis.

[107] Ng J.K., Wang W., Liu W.T., Chong S.S. (2010). Spatially addressable bead-based biosensor for rapid detection of beta-thalassemia mutations. Anal. Chim. Acta.

[108] Ikami M., Kawakami A., Kakuta M., Okamoto Y., Kaji N., Tokeshi M., Baba Y. (2010). Immuno-pillar chip: A new platform for rapid and easy-to-use immunoassay. Lab Chip.

[109] Peredy T.R., Powers R.D. (1997). Bedside diagnostic testing of body fluids. Am. J. Emerg. Med..

[110] Altieri M.F., Camarca M. (2001). Point of care testing. Clin. Pediatr. Emerg. Med..

[111] Christodoulides N., Dharshan P., Wong J., Floriano P.N., Neikirk D., McDevitt J.T. (2007). A microchip-based assay for interleukin-6. Methods Mol. Biol..

[112] Christodoulides N., Mohanty S., Miller C.S., Langub M.C., Floriano P.N., Dharshan P., Ali M.F., Bernard B., Romanovicz D., Anslyn E. (2005). Application of microchip assay system for the measurement of c-reactive protein in human saliva. Lab Chip.

[113] Christodoulides N., Tran M., Floriano P.N., Rodriguez M., Goodey A., Ali M., Neikirk D., McDevitt J.T. (2002). A microchip-based multianalyte assay system for the assessment of cardiac risk. Anal. Chem..

[114] Goodey A., Lavigne J.J., Savoy S.M., Rodriguez M.D., Curey T., Tsao A., Simmons G., Wright J., Yoo S.J., Sohn Y. (2001). Development of multianalyte sensor arrays composed of chemically derivatized polymeric microspheres localized in micromachined cavities. J. Am. Chem. Soc..

[115] Rubina A.Y., Dementieva E.I., Stomakhin A.A., Darii E.L., Pan'kov S.V., Barsky V.E., Ivanov S.M., Konovalova E.V., Mirzabekov A.D. (2003). Hydrogel-based protein microchips: Manufacturing, properties, and applications. Biotechniques.

[116] Zubtsov D.A., Savvateeva E.N., Rubina A.Y., Pan'kov S.V., Konovalova E.V., Moiseeva O.V., Chechetkin V.R., Zasedatelev A.S. (2007). Comparison of surface and hydrogel-based protein microchips. Anal. Biochem..

[117] Chou J., Lennart A., Wong J., Ali M.F., Floriano P.N., Christodoulides N., Camp J., McDevitt J.T. (2012). Modeling analyte transport and capture in porous bead sensors. Anal. Chem..

[118] Sanhai W.R., Sakamoto J.H., Canady R., Ferrari M. (2008). Seven challenges for nanomedicine. Nat. Nanotechnol..

[119] Seong G.H., Crooks R.M. (2002). Efficient mixing and reactions within microfluidic channels using microbead-supported catalysts. J. Am. Chem. Soc..

[120] Liu C., Schrlau M.G., Bau H.H. (2009). Single bead-based electrochemical biosensor. Biosens. Bioelectron..

[121] Nazir A., Schroën K., Boom R. (2010). Premix emulsification: A review. J. Membr. Sci..

[122] De Mello A.J., Beard N. (2003). Focus. Dealing with “real” samples: Sample pre-treatment in microfluidic systems. Lab Chip.

[123] Gervais T., Jensen K.F. (2006). Mass transport and surface reactions in microfluidic systems. Chem. Eng. Sci..

[124] Zhan W., Seong G.H., Crooks R.M. (2002). Hydrogel-based microreactors as a functional component of microfluidic systems. Anal. Chem..

[125] Rubina A.Y., Pan'kov S.V., Dementieva E.I., Pan'kov D.N., Butygin A.V., Vasiliskov V.A., Chudinov A.V., Mikheikin A.L., Mikhailovich V.M., Mirzabekov A.D. (2004). Hydrogel drop microchips with immobilized DNA: Properties and methods for large-scale production. Anal. Biochem..

[126] Khademhosseini A., Yeh J., Jon S., Eng G., Suh K.Y., Burdick J.A., Langer R. (2004). Molded polyethylene glycol microstructures for capturing cells within microfluidic channels. Lab Chip.

[127] Sung W.C., Chen H.H., Makamba H., Chen S.H. (2009). Functionalized 3d-hydrogel plugs covalently patterned inside hydrophilic poly(dimethylsiloxane) microchannels for flow-through immunoassays. Anal. Chem..

[128] Pernodet N., Maaloum M., Tinland B. (1997). Pore size of agarose gels by atomic force microscopy. Electrophoresis.

[129] Wang R.Y., Liu X.Y., Narayanan J., Xiong J.Y., Li J.L. (2006). Architecture of fiber network: From understanding to engineering of molecular gels. J. Phys. Chem. B.

[130] Larsson P.O., Gustavsson P.E., Axelsson A. (1998). Direct measurement of intraparticle fluid velocity in superporous agarose beads. J. Mol. Recognit..

[131] Thompson J.A., Bau H.H. (2010). Microfluidic, bead-based assay: Theory and experiments. J. Chromatogr. B.

[132] Thompson J., Bau H. (2012). Porous bead-based microfluidic assay: Theory and confocal microscope imaging. Microfluid. Nanofluid..

[133] Chen L., Chen Z.T., Wang J., Xiao S.J., Lu Z.H., Gu Z.Z., Kang L., Chen J., Wu P.H., Tang Y.C. (2009). Gel-pad microarrays templated by patterned porous silicon for dual-mode detection of proteins. Lab Chip.

[134] Arenkov P., Kukhtin A., Gemmell A., Voloshchuk S., Chupeeva V., Mirzabekov A. (2000). Protein microchips: Use for immunoassay and enzymatic reactions. Anal. Biochem..

[135] Thompson J.A., Bau H.H. (2011). Pulsating bead-based assay. Anal. Chem..

[136] Chou J., Kulla E., Simmons G.W., Floriano P.N., Christodoulides N., McDevitt J.T. (2012). Enhancement of performance in bead based microfluidics: Effects of geometry. Lab Chip.

[137] Coulson J.M., Richardson J.F., Backhurst J.R., Harker J.H. (1991). Chemical Engineering.

[138] Chou J., Li L.E., Christodoulides N., Floriano P.N., McDevitt J.T. (2012). Effects of sample delivery on analyte capture in porous bead sensors. Lab Chip.

[139] Gustavsson P.E., Axelsson A., Larsson P.O. (1998). Direct measurements of convective fluid velocities in superporous agarose beads. J. Chromatogr. A.

[140] Gustavsson P.E., Axelsson A., Larsson P.O. (1999). Superporous agarose beads as a hydrophobic interaction chromatography support. J. Chromatogr. A.

[141] Du K.F., Bai S., Dong X.Y., Sun Y. (2010). Fabrication of superporous agarose beads for protein adsorption: Effect of CaCO_3_ granules content. J. Chromatogr. A.

[142] Gottschalk I., Gustavsson P.E., Ersson B., Lundahl P. (2003). Improved lectin-mediated immobilization of human red blood cells in superporous agarose beads. J. Chromatogr. B.

[143] Yang Y., Nam S.-W., Lee N.Y., Kim Y.S., Park S. (2008). Superporous agarose beads as a solid support for microfluidic immunoassay. Ultramicroscopy.

[144] Zubtsov D.A., Ivanov S.M., Rubina A.Y., Dementieva E.I., Chechetkin V.R., Zasedatelev A.S. (2006). Effect of mixing on reaction-diffusion kinetics for protein hydrogel-based microchips. J. Biotechnol..

[145] Sia S.K., Kricka L.J. (2008). Microfluidics and point-of-care testing. Lab Chip.

[146] Schully S.D., Benedicto C.B., Gillanders E.M., Wang S.S., Khoury M.J. (2011). Translational research in cancer genetics: The road less traveled. Public Health Genomics.

[147] Weigum S.E., Floriano P.N., Christodoulides N., McDevitt J.T. (2007). Cell-based sensor for analysis of egfr biomarker expression in oral cancer. Lab Chip.

[148] Weigum S.E., Floriano P.N., Redding S.W., Yeh C.K., Westbrook S.D., McGuff H.S., Lin A., Miller F.R., Villarreal F., Rowan S.D. (2010). Nano-bio-chip sensor platform for examination of oral exfoliative cytology. Cancer Prev. Res..

[149] Yeh C.K., Christodoulides N.J., Floriano P.N., Miller C.S., Ebersole J.L., Weigum S.E., McDevitt J., Redding S.W. (2010). Current development of saliva/oral fluid-based diagnostics. Tex. Dent. J..

[150] Christodoulides N., Floriano P.N., Sanchez X., Li L., Hocquard K., Patton A., Muldoon R., Miller C.S., Ebersole J.L., Redding S. (2012). Programmable bio-nanochip technology for the diagnosis of cardiovascular disease at the point of care. Methodist DeBakey Cardiovasc. J..

[151] Long G.L., Winefordner J.D. (1983). Limit of detection a closer look at the iupac definition. Anal. Chem..

[152] MacDougall D., Crummett W.B. (1980). Guidelines for data acquisition and data quality evaluation in environmental chemistry. Anal. Chem..

[153] McNaught A.D., Wilkinson A. (1997). Compendium of Chemical Terminology.

[154] US Department of Health and Human Services National Health Expenditures 2009 Highlights.

[155] Group T.L. (2005). The Value of Diagnostics Innovation, Adoption and Diffusion into Health Care.

[156] Gaidos S. A Spitting Image of Health.

